# Lumbar degenerative disease treated by percutaneous endoscopic transforaminal lumbar interbody fusion or minimally invasive surgery-transforaminal lumbar interbody fusion: a case-matched comparative study

**DOI:** 10.1186/s13018-021-02841-4

**Published:** 2021-11-27

**Authors:** You-Di Xue, Wen-Bo Diao, Chao Ma, Jie Li

**Affiliations:** 1 Department of Orthopaedics, Xuzhou Central Hospital, Xuzhou Clinical School of Xuzhou Medical University, Xuzhou Clinical College of Nanjing Medical University, 199 Jiefang South Road, Xuzhou, 221009 Jiangsu Province People’s Republic of China; 2 Department of Orthopaedics, Zhoukou Orthopedic Hospital, Zhoukou, 466000 Henan People’s Republic of China

**Keywords:** Percutaneous endoscopic transforaminal lumbar interbody fusion, PETLIF, Minimally invasive surgery-transforaminal lumbar interbody fusion, MISTLIF, Degenerative lumbar disease, Spinal surgery

## Abstract

**Purpose:**

This study aimed to evaluate the clinical efficacy and imaging results of percutaneous endoscopic transforaminal lumbar interbody fusion (PETLIF) through comparing it with minimally invasive surgery-transforaminal lumbar interbody fusion (MISTLIF).

**Materials and methods:**

We performed a retrospective analysis on patients with lumbar degenerative disease treated by PETLIF or MISTLIF from September 2017 to January 2019, and the patients were divided into two groups: the PETLIF group and the MISTLIF group. The clinical and imaging parameters of the two groups were evaluated.

**Results:**

There was no significant difference between the two groups in complication rate. The operative time in the PETLIF group was significantly less than that in the MISTLIF group. Compared with those before operation, the postoperative VAS-L and VAS-B scores were significantly improved after operation in the both groups. In addition, the postoperative VAS-B score of the PETLIF group was significantly lower than that of the MISTLIF group. At the last follow-up, there was no significant difference between the two groups in the VAS-L score, VAS-B score, ODI score, and bony fusion rate.

**Conclusions:**

Both PETLIF and MISTLIF could achieve satisfactory clinical outcomes in the treatment of lumbar degenerative disease, but our study suggested that PETLIF had less damage, rapid recovery after operation, and short discharge time.

**Supplementary Information:**

The online version contains supplementary material available at 10.1186/s13018-021-02841-4.

## Introduction

Spinal endoscopic surgery, as a minimally invasive surgery, has developed rapidly in recent years. The techniques of endoscopic decompression and discectomy have been fully developed and widely used for the treatment of lumbar disc herniation and lumbar spinal stenosis [1]. However, the spinal canal decompression for the treatment of some diseases such as lumbar spondylolisthesis, lumbar instability and intractable back pain still cannot achieve satisfactory clinical outcomes. It is considered that the restoration of spinal alignment and the reconstruction of spinal stability via lumbar spinal fusion are necessary for excellent long-term clinical outcomes of these diseases. Lumbar spinal fusion has been used for hundreds of years, and it can rebuild the spinal stability while decompressing spinal canal stenosis. Posterior lumbar fusion surgery, as the earliest surgical method of spinal fusion, is widely used, but it will inevitably cause ligaments and facet joints damage, and in addition extensive soft tissue dissection usually occurs, with slow recovery and substantial complications. In order to reduce iatrogenic injuries and complications, various improved or new techniques have emerged one after anther, such as transforaminal lumbar interbody fusion, anterior lumbar interbody fusion, oblique lumbar interbody fusion, and extreme lateral interbody fusion [2], and they are committed to achieve fusion and nerve decompression with minimal surgical trauma and preserving normal tissue structure. Percutaneous endoscopic interbody fusion through transforaminal approach is a minimally invasive surgery based on the spinal endoscopic technique. At present, several studies have suggested that this technique can significantly reduce surgical trauma and blood loss, with short hospital stay and quick recovery [3–5].

This study compared the clinical efficacy, imaging data, and functional results of percutaneous endoscopic transforaminal lumbar interbody fusion (PETLIF) with those of minimally invasive surgery-transforaminal lumbar interbody fusion (MISTLIF) to evaluate the clinical outcomes of PETLIF.

## Materials and methods

This study was conducted in accordance with the declaration of Helsinki. This study was conducted with approval from the Ethics Committee of Xuzhou Central Hospital. Written informed consent was obtained from the participants. This paper was prepared guided by the STROBE checklist (Additional file 1).

### Sample size calculation

The calculation of sample size was based on the preliminary results of this study. The intraoperative bleeding of the single-segment MISTLIF was 150 ml, and that of the single-segment PETLIF was 70 mL. The value of α was set to 0.05, and the power to 0.80. When the sample size of each group was 20, the difference between the two groups was statistically significant.

### Inclusion criteria

Cases with single-segment degenerative diseases of the lumbar spine were confirmed by imaging examinations, including first-degree degenerative lumbar spondylolisthesis, lumbar disc herniation combined with lumbar instability, and lumbar spinal stenosis; cases signed the informed consent to participate in the study; cases had complete clinical and imaging data; cases were followed up for at least 12 months.

### Exclusion criteria

Cases experienced severe central spinal stenosis, spondylolisthesis of degree II or higher, severe osteoporosis, a history of lumbar spine surgery, other neurological diseases, or mental abnormalities; cases did not have complete clinical or imaging data; cases were followed up for less than 12 months.

### Subjects

From September 2017 to January 2019, our department used PETLIF to treat 20 patients with lumbar degenerative disease. All these patients completed at least 12 months of follow-up and were included in the PETLIF group of this study. At the same time, another 83 patients with lumbar degenerative disease were treated by MISTLIF in our department, of which the 20 cases, matching with the PETLIF group in age, sex, course of disease, and affected level, were included in the MISTLIF group of this study.

### Evaluating indicators

The following data of the PETLIF group and the MISTLIF group were recorded: surgical segment, operation time, estimated blood loss, total incision length, complications, and hospital stay. The visual analog scale (VAS) was used to evaluate low back pain (VAS-B) and leg pain (VAS-L), and the Oswestry Disability Index (ODI) was used to evaluate the clinical outcomes of the two groups. The VAS-B score, VAS-L score, and ODI score were compared between the two groups before the operation, after the operation, at the 12-month follow-up, and at the last follow-up, respectively. Radiographic examinations included lumbar X-ray films, and lumbar three-dimensional computed tomography scans (3D-CT). The X-ray films were performed before surgery, after surgery, at the 12-month follow-up, and at the last follow-up; meanwhile, 3D-CT scans were performed at 12 months after surgery and at the last follow-up.

According to the interbody fusion criteria introduced by Proietti et al. [6] and Berjano et al. [7], the interbody fusion was obtained when one or more bony bridges were present in the intervertebral space confirmed by 3D-CT in this study. The pseudarthrosis was identified when the bone bridge was absent, no bone graft was visible in the cage, and radiolucency was seen at interfaces, or bone resorption surrounding the cage or the screws.

### Surgical technique

**Fig. 1:**
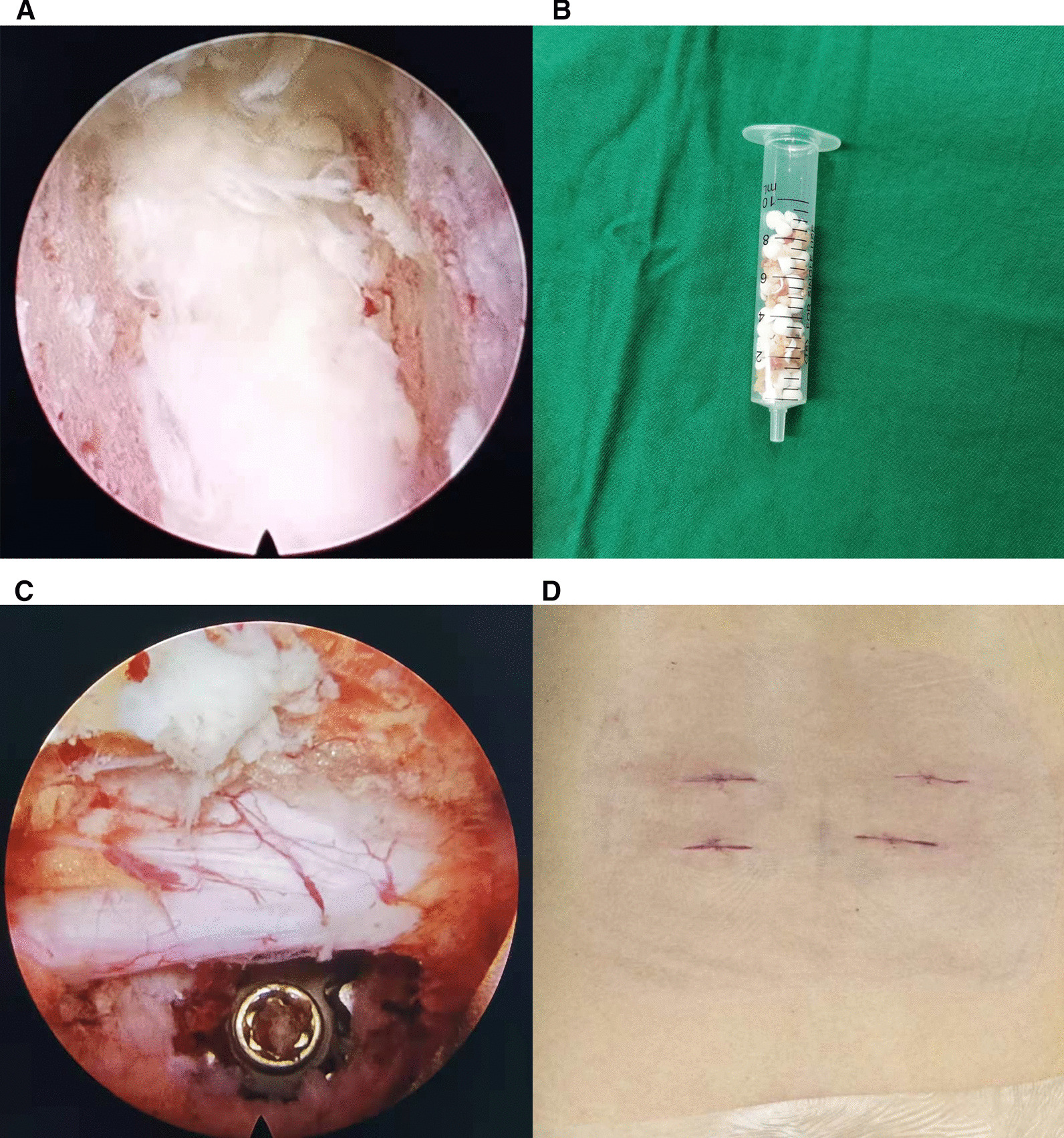
**A** Intraoperative endoscopic image shows excellent bone graft bed in the intervertebral space. **B** The mixture of autogenous bone and allograft bone has been prepared for bone graft. **C** Intraoperative endoscopic image shows decompression of central canal and lateral recess, with good implantation of cage. **D** Skin incisions for the PETLIF procedure

In the PETLIF group, patients received general anesthesia and turned to the prone position on the radiolucent operative bed. Under the image intensifier, the working channel was constructed according to the Kambin principle, and the spinal endoscopy was implanted. Then in order to enlarge the intervertebral foramen, the facet joint was confirmed and partial superior facetectomy was performed. The ligamentum flavum was removed, and the dural sac and nerve roots were exposed and protected. Then, the nucleus pulposus and cartilage endplate were removed (Fig. 1A), and a percutaneous titanium cage filled with a mixture of the autologous and allogeneic bone was implanted in the intervertebral space through the endoscopic tube (Fig. 1B, C). After hemostasis, the nerve tissue and the cage were observed again, the instruments were pulled out, and the wound was sutured. Finally, a minimally invasive spinal internal fixation system was used to insert percutaneous pedicle screws, and the incisions were sutured layer by layer (Fig. 1D).

The preoperative preparations in the MISTLIF group were the same as those in the PETLIF group. Under the image intensifier, the operative level was confirmed and the position of the pedicle on the body surface was marked. A longitudinal incision on the symptomatic side was made about 3–5 cm from the middle line between the caudal and the cranial pedicle at the affected level. The space between longissimus and multifidus was separated by sequential dilation to expose the vertebral laminae and the facet joint. Then, the decompression procedure was performed using osteotome and Kerrison rongeurs, and the nerve roots were exposed and protected. After the intervertebral space was prepared, a titanium cage filled with autologous bone and allogeneic bone was implanted in the intervertebral space. Under the guidance of fluoroscopy, the percutaneous minimally invasive pedicle screw insertion procedure was performed. Finally, the drainage was placed, and the incision was sutured layer by layer.

### Postoperative management

All patients in the both groups received prophylactic antibiotics for 24 h after operation. The patients in the PETLIF group were discharged from the hospital on the second day after operation, and their off-bed activities began as early as 6 h after operation. In the MISTLIF group, the drainage tube was removed 24–48 h after surgery, and the patients were allowed to be ambulant and discharged from the hospital.

### Statistical analysis

SPSS11.0 was used for statistical analysis, the quantitative data were analyzed by *T* test, and the qualitative data were analyzed by Chi-square test. If the *P* value was less than 0.05, the difference was considered to be statistically significant.

## Results

In the PETLIF group, there were 11 males and 9 females, with an average age of 46.3 years old (ranging from 26 to 67 years old), 6 cases with L3-4 involvement, and 14 cases with L4-5 involvement. In the MISTLIF group, there were 12 males and 8 females, with an average age of 47.1 years (ranging from 28 to 64 years), 5 cases with L3-4 involvement, and 15 cases with L4-5 involvement. There were no statistical differences in the demographic data and the involvement level between the two groups (Table 1).

**Table 1:** Demographic and baseline characteristics of all patients

Items	The PETLIF group (*n* = 20)	The MISTLIF group (*n* = 20)	*P* value
Age (years)	46.3 ± 17.2	48.4 ± 13.6	0.763
Sex			0.539
Male	11	12	
Female	9	8	
Disease course (months)	27.4 ± 8.7	28.2 ± 6.3	0.571
Disease level			0.942
L3-4	6	5	
L4-5	14	15	

The average follow-up time was 16.1 ± 6.3 months (ranging from 12 to 23 months) in the PETLIF group and 15.8 ± 7.2 months (ranging from 13 to 24 months) in the MISTLIF group; the average operation time was 140.3 ± 35.6 min in the PETLIF group and 170.6 ± 54.8 min in the MISTLIF group; the estimated blood loss was 65.6 ± 15.3 ml in the PETLIF group and 140.5 ± 21.5 ml in the MISTLIF group. The operation time and estimated blood loss in the PETLIF group were significantly less than those in the MISTLIF group (Table 2).

**Table 2:** Surgical- and clinical-related data of both groups

	The PETLIF group (*n* = 20)	The MISTLIF group (*n* = 20)	*P* value
Operation time (min)	140.3 ± 35.6	170.6 ± 54.8	0.000
Estimated blood loss (ml)	65.6 ± 15.3	140.5 ± 21.5	0.000
Total incision length (cm)	5.3 ± 0.8	7.8 ± 2.3	0.000
Length of stay (d)	2.4 ± 1.6	4.5 ± 2.1	0.000
Follow-up time (months)	16.1 ± 6.3	15.8 ± 7.2	0.931

In the PETLIF group, the average VAS-B score was 7.3 ± 1.2 before surgery, which significantly decreased to 2.3 ± 0.9 (*p* = 0.000) after surgery and to 1.9 ± 1.1 (*p* = 0.000) at the last follow-up, and the average VAS-L score was 7.2 ± 0.8 before surgery, which significantly decreased to 2.8 ± 1.0 (*p* = 0.000) after surgery and to 1.7 ± 1.2 (*p* = 0.000) at the last follow-up. In the MISTLIF group, the average VAS-B score was 7.0 ± 0.8 before surgery, which significantly decreased to 4.1 ± 1.2 (*p* = 0.000) after surgery and to 1.8 ± 0.7 (*p* = 0.000) at the last follow-up, and the average VAS-L score was 7.2 ± 1.1 before surgery, which significantly decreased to 2.7 ± 0.6 (*p* = 0.000) after surgery and to 1.6 ± 0.9 (*p* = 0.000) at the last follow-up. The VAS scores before surgery and at the last follow-up were very similar in the both groups; however, the postoperative VAS-B score in the PETLIF group was significantly lower than that in the MISTLIF group (*p* = 0.000). The average ODI score of the PETLIF group significantly improved from 85.1 ± 7.3 before surgery to 41.7 ± 9.8 at the 3-month follow-up and to 30.4 ± 7.3 at the last follow-up (*p* = 0.000). The average ODI score of the MISTLIF group significantly improved from 84.4 ± 8.3 before surgery to 45.5 ± 11.3 at the 3-month follow-up and to 31.5 ± 7.8 at the last follow-up (*p* = 0.000). There was no statistically significant difference between the two groups at each follow-up point (Table 3).

**Table 3:** Preoperative and postoperative outcomes of VAS and ODI scores for both groups

	The PETLIF group (*n* = 20)	The MISTLIF group (*n* = 20)	*P* value
VAS-B			
Preoperative	7.3 ± 1.2	7.0 ± 0.8	0.847
Postoperative	2.3 ± 0.9	4.1 ± 1.2	0.000
3-month postoperative	2.1 ± 0.9	2.3 ± 1.1	0.529
At the last follow-up	1.9 ± 1.1	1.8 ± 0.7	0.731
VAS-L			
Preoperative	7.2 ± 0.8	7.2 ± 1.1	0.692
Postoperative	2.8 ± 1.0	2.7 ± 0.6	0.739
3-month postoperative	2.0 ± 1.3	1.9 ± 0.6	0.581
At the last follow-up	1.7 ± 1.2	1.6 ± 0.9	0.216
ODI			
Preoperative	85.1 ± 7.3	84.4 ± 8.3	0.539
3-month postoperative	41.7 ± 9.8	45.5 ± 11.3	0.213
At the last follow-up	30.4 ± 7.3	31.5 ± 7.8	0.192

**Fig. 2:**
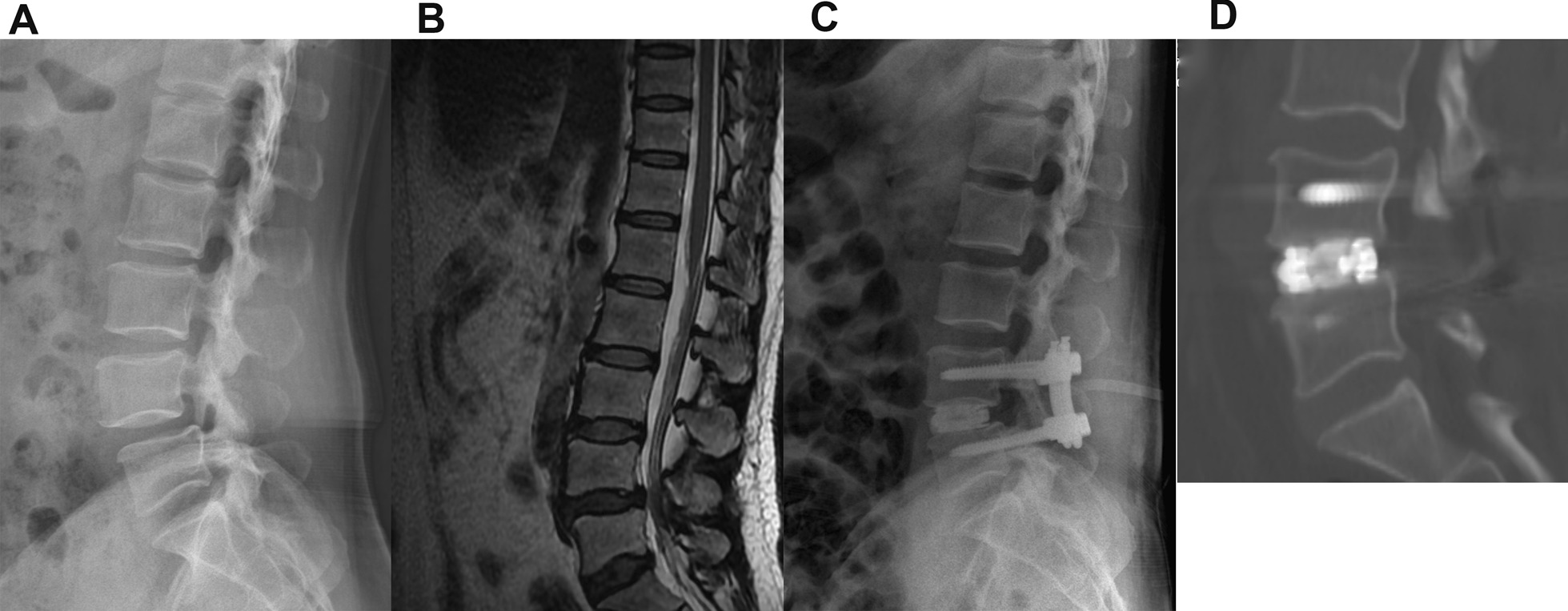
A 54-year-old male with L4-5 spinal canal stenosis, who was treated with percutaneous endoscopic transforaminal lumbar interbody fusion and percutaneous pedicle screw fixation. **A** Preoperative lateral radiograph (**A**) and MRI (**B**) show degenerative spondylolisthesis and canal stenosis with narrowed disc space at L4-5. **C** Postoperative lateral radiograph shows excellent screws and cage location with improved disc space height. **D** Sagittal CT obtained at the last follow-up duration (15 months after operation) shows interbody bony fusion

According to the interbody fusion criteria introduced by Luca Proietti and Pedro Berjano, at the 12-month follow-up, 18 of 20 patients in the PETLIF group achieved bone fusion, with the fusion rate of 90%, while 17 of 20 patients in the MISTLIF patients achieved bone fusion, with the fusion rate of 85%. All patients in the two groups obtained bone fusion at the last follow-up (Fig. 2A–D).

In the MISTLIF group, two patients experienced hypoesthesia of the lower extremity innervation, and their symptoms were relieved after treatment. For them, one’s symptoms were relieved at the one-month follow-up, and the other one’s symptoms disappeared at the 3-month follow-up duration. One patient underwent cerebrospinal fluid leakage after operation, requiring prolonged duration for extubation, and body position change. In addition, one patient had delayed incision healing without superficial infection.

In the PETLIF group, two patients had headaches after surgery, of whom one with mild symptom achieved complete relief after oral administration of nonsteroidal anti-inflammatory drugs, and the other one had a severe headache, with a high-density shadow in part of the sulcus and cistum observed from the brain CT, thereby being diagnosed with subarachnoid hemorrhage and without intracranial aneurysm, vascular malformation and venous thrombosis confirmed by magnetic resonance angiography (MRA) of the brain. The latter was treated with bed rest, analgesia, mannitol, and nimodipine, and the symptoms were alleviated. Three months after the operation, the CT scan showed the hematoma was completely absorbed.

## Discussion

Lumbar spinal fusion is the most common treatment method of lumbar spine disorders. From the early posterolateral fusion, various spinal fusion procedures have gradually appeared. On the one hand, the continuous improvements of surgical procedures suggest the deeper understanding of the anatomical structure and the instrument improvement; on the other hand, it also shows that there is no ideal fusion technique at present. Posterior lumbar interbody fusion (PLIF) surgery could achieve interbody spinal fusion while decompressing the spinal canal directly and thoroughly. However, PLIF can bring great damage to the paravertebral soft tissue and bone structure and can greatly interfere with dural sac and nerve roots. TLIF can reduce the iatrogenic injuries and avoid excessive traction on nerve tissue. However, for the TLIF, most of the ipsilateral facet joints and ligamentum flavum need to be removed before decompression and implantation of the fusion cage. ALIF, OLIF, and LLIF entering intervertebral space from the lateral or anterior side can avoid the destruction of posterior muscles and osseous structures, without encroaching on the spinal canal. However, the spinal surgeons are not familiar with the anatomical structure, and thus, the injuries of the important organs, such as the abdominal cavity and large blood vessels, might occur during the operation [8]. The decompression of these procedures is indirect, with limited indications, and sometimes it might be necessary to change the body position to obtain auxiliary pedicle screw fixation. In 2002, Foley et al. [9] proposed MISTLIF to reduce the damage of muscle and bone structure. This technique uses a dilation system to establish a surgical access through the intramuscular space, and then, the decompression and interbody fusion are performed from the intervertebral foramen. However, the working channel is long and narrow, the anatomical structure is difficult to identify, and the operation might be inconvenient, so the early learning curve is steep, and the incidence of complications is high. Nerve injuries, surgical site infection, hematoma, and the leakage of cerebrospina are complications commonly reported in the literature [10]. In addition, muscle atrophy and ischemic necrosis might occur due to the continuous squeezing of the working channel [11]. In recent years, the spinal endoscopy has been widely used in the lumbar spine and achieved satisfactory clinical outcomes. Besides, some surgeons have begun to perform lumbar fusion under the endoscope [3, 4, 12] and have achieved satisfactory clinical outcomes.

In this study, the two groups both achieved satisfactory clinical results, and there were no serious complications in the interbody fusion, indicating that these two types of procedures were safe and effective in the treatment of lumbar degenerative diseases.

The average length of hospital stay in the PETLIF group (2.4 days) was shorter than that in the MISTLIF group (4.5 days), with a significant difference, which indicated that the PETLIF surgery could promote the recovery and shorten the hospital stay due to less surgical trauma.

The VAS-L score in the two groups significantly decreased after operation, indicating that PETLIF and MISTLIF both can achieve complete spinal canal decompression. The VAS-B score in the two groups also significantly decreased after operation, but the postoperative VAS-B score in the PETLIF group was significantly lower than that in the MISTLIF group, for which the reason might be that the PETLIF procedure caused less surgical trauma to the soft tissue and bone structure than the MISTLIF procedure.

As we all know, bone grafts play an important role in interbody fusion. Studies [13, 14] suggested that bone grafts and intervertebral cages should cover at least 30% of the surface area of the endplate to provide sufficient stability while allowing early movement. Autologous bone has always been the gold standard for bone grafts. Traditional fusion surgery can obtain enough autologous bone for grafting, but the amount of autologous bone collected by PETLIF surgery is insufficient, which cannot meet the needs of intervertebral bone grafting. Various sources of bone grafts have already been reported in the literature [15]. We implanted bone harvest from facet joint mixed with allogeneic bone into the cage and obtained satisfactory bone fusion during the follow-up period.

Nerve root injury is a serious complication of endoscopic surgery, especially the injury of the exiting nerve roots, which deserves special attention [16]. The intervertebral foraminal stenosis and prolonged operation time are the main risk factors for nerve root injury [17]. Obvious intervertebral stenosis and level II of spondylolisthesis should be regarded as relative contraindications, and the reduction of intervertebral height and changes of local anatomical structure result in Kambin triangle changes, which makes it difficult to establish working channels and puts the nerve roots in danger [18]. Anatomical studies have shown that the exiting nerve roots are adjacent to the upper facet joints at the disc level, so the removal of part facet joint to expand Kambin triangle is a key step to reduce nerve root injuries [19]. In addition, the long operation time is another high-risk factor that leads to nerve injuries. Partial resection of the upper facet joint can achieve lateral recess decompression while expanding the surgical channel, which can reduce the operation time and the risk of nerve damage. Moreover, skilled surgical techniques can significantly reduce the operation time and the impact of instruments on the existing nerve roots.

Postoperative headache is a common complication of spinal endoscopic procedure. Endoscopic surgery requires continuous infusion of normal saline into the spinal canal, which leads to an increase in epidural pressure and changes in cerebrospinal fluid. Higuchi et al. [20] used MRI to study the degree and duration of compression of the dural sac after the injection of normal saline into epidural space, and the results showed that changes in the cerebrospinal fluid (CSF) volume were related to the volume of normal saline. The compression degree lasted at least 30 min after saline injection. Joh et al. [21] measured cervical epidural pressure in 28 patients undergoing spinal endoscopic surgery and found that with the continuous epidural perfusion, the epidural pressure continued to rise, and high pressure was closely related to intraoperative cervical pain. Choi et al. [22] reported that during spinal endoscopic surgery, elevated epidural pressure can cause cervical pain and even induce epilepsy. In this study, two patients had headaches, of which one had mild symptoms, and the symptoms were relieved after symptomatic treatment, which might be caused by the increased epidural pressure by the continuous perfusion, and the other one had severe headache, which could not be alleviated by the symptomatic treatment. Brain CT scan showed high-density shadows in the brain sulcus and cistern, and MRA excluded the lesions such as intracranial aneurysm, vascular malformation, and venous thrombosis leading to spontaneous intracranial hemorrhage. Therefore, subarachnoid hemorrhage was considered. The reasons might be as follows: firstly, after epidural perfusion of saline, the increased intracranial pressure might lead to passive expansion and congestion of cerebral blood vessels, which might increase the permeability of blood vessels and blood cell exudation and eventually induce intracranial hemorrhage; secondly, after the dural sac is compressed, it might cause the occlusion of the epidural vein, resulting in increased intracranial venous pressure, causing the intracranial vein to dilate and congest and eventually rupture and hemorrhage.

This study has several limitations. First of all, the sample size is small and the follow-up time is short. In the future, clinical observations with multiple centers, large sample size, and long-term follow-up are needed to evaluate the clinical efficacy of PETLIF in the treatment of lumbar degenerative disease. Secondly, there are many minimally invasive surgical procedures for lumbar fusion, including OLIF, ALIF, and other anterior lumbar fusion, but only MISTLIF was used as the control group in this study. Therefore, it is necessary to compare with other procedures in the future. Last but not least, this study is a retrospective case–control study, and a higher-level evidence is required in the future.

## Conclusions

In summary, PETLIF and MISTLIF in the treatment of lumbar degenerative disease both could achieve satisfactory clinical outcomes, but our study suggested that PETLIF had less damage, rapid recovery after operation, and short discharge time.

## Supplementary Information

**Additional file 1**. STROBE Statement—checklist of items of observational studies.

## Data Availability

The datasets used and/or analyzed during the current study are available from the corresponding author on reasonable request.

## Abbreviations

PETLIF: Percutaneous endoscopic transforaminal lumbar interbody fusion
MISTLIF: Minimally invasive surgery-transforaminal lumbar interbody fusion
TLIF: Transforaminal lumbar interbody fusion
ALIF: Anterior lumbar interbody fusion
OLIF: Oblique lumbar interbody fusion
XLIF: Extreme lateral interbody fusion
VAS: Visual analog scale
VAS-B: Visual analog scale (VAS) was used to evaluate low back pain
VAS-L: Visual analog scale (VAS) was used to evaluate leg pain
ODI: Oswestry Disability Index
3D-CT: Three-dimensional computed tomography scans
CTA: Computed tomography angiography
MRA: Magnetic resonance angiography
PLF: Posterolateral fusion
PLIF: Posterior lumbar interbody fusion
ALIF: Anterior lumbar interbody fusion
LLIF: Lateral lumbar interbody fusion
