# Correction to: Lumbar degenerative disease treated by percutaneous endoscopic transforaminal lumbar interbody fusion or minimally invasive surgery-transforaminal lumbar interbody fusion: a case-matched comparative study

**DOI:** 10.1186/s13018-022-02943-7

**Published:** 2022-02-22

**Authors:** You-Di Xue, Wen-Bo Diao, Chao Ma, Jie Li

**Affiliations:** 1 Department of Orthopaedics, Xuzhou Central Hospital, Xuzhou Clinical School of Xuzhou Medical University, Xuzhou Clinical College of Nanjing Medical University, 199 Jiefang South Road, Xuzhou, 221009 Jiangsu Province People’s Republic of China; 2 Department of Orthopaedics, Zhoukou Orthopedic Hospital, Zhoukou, 466000 Henan People’s Republic of China

## Correction to: Journal of Orthopaedic Surgery and Research (2021) 16:696 10.1186/s13018-021-02841-4

Following publication of the original article [1], an error was identified in the abstract section.

The updated abstract is given below and the changes have been highlighted in **bold typeface**.

"There was no significant difference between the two groups in complication rate. The operative time in the PETLIF group was significantly less than that in the MISTLIF group".

